# Defining the Emerging Blood System During Development at Single-Cell Resolution

**DOI:** 10.3389/fcell.2021.660350

**Published:** 2021-05-12

**Authors:** Göran Karlsson, Mikael N. E. Sommarin, Charlotta Böiers

**Affiliations:** Division of Molecular Hematology, Lund Stem Cell Center, Lund University, Lund, Sweden

**Keywords:** single-cell RNA sequencing, embryonic haematopoiesis, single-cell ATAC sequencing, lineage hierarchy, single-cell genomics, endothelial to hematopoietic transition (EHT), hematopoietic stem cells (HSC)

## Abstract

Developmental hematopoiesis differs from adult and is far less described. In the developing embryo, waves of lineage-restricted blood precede the ultimate emergence of definitive hematopoietic stem cells (dHSCs) capable of maintaining hematopoiesis throughout life. During the last two decades, the advent of single-cell genomics has provided tools to circumvent previously impeding characteristics of embryonic hematopoiesis, such as cell heterogeneity and rare cell states, allowing for definition of lineage trajectories, cellular hierarchies, and cell-type specification. The field has rapidly advanced from microfluidic platforms and targeted gene expression analysis, to high throughput unbiased single-cell transcriptomic profiling, single-cell chromatin analysis, and cell tracing—offering a plethora of tools to resolve important questions within hematopoietic development. Here, we describe how these technologies have been implemented to address a wide range of aspects of embryonic hematopoiesis ranging from the gene regulatory network of dHSC formation via endothelial to hematopoietic transition (EHT) and how EHT can be recapitulated *in vitro*, to hematopoietic trajectories and cell fate decisions. Together, these studies have important relevance for regenerative medicine and for our understanding of genetic blood disorders and childhood leukemias.

## Introduction

The well-characterized, gradual maturation of hematopoietic stem cells (HSCs) into functional blood and immune cells serves as a conceptual model for stem-cell-related processes like hierarchical organization, lineage commitment, cell fate decision, and malignant transformation. Historically, breakthroughs in our understanding of hematopoiesis have been intimately correlated with technological advances (Doulatov et al., 2012; Jacobsen and Nerlov, 2019). Accordingly, the current era of single-cell genomics has already had fast and valuable impact even on the most central views of hematopoietic differentiation, questioning the dogma of the classical, stepwise lineage commitment of hematopoiesis (Laurenti and Gottgens, 2018; Jacobsen and Nerlov, 2019).

**FIGURE 1:**
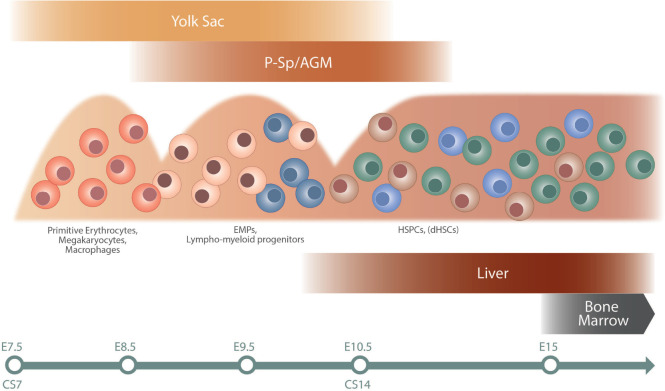
Schematic overview of hematopoietic development. The different waves of hematopoietic cells are illustrated, as well as site of emergence (top) and colonization (bottom). The timeline at the bottom indicates approximate time in mouse (E) and human (CS). The first primitive wave of hematopoietic cells emerges in the yolk sac at approximately E7 in mouse and CS7 in human. This is followed by a second wave of EMPs and lymphoid/lympho-myeloid progenitors in mouse, a wave not fully characterized in human (Ivanovs et al., 2017). The third wave initiates in the AGM region and gives rise to HSPC and dHSCs. Progenitors from the second and third waves colonize the fetal liver. The bone marrow is colonized later in development, around E15 in mouse, and becomes the dominating hematopoietic niche around birth. AGM, aorta–gonad–mesonephros; CS, Carnegie stage; E, embryonic day; EMPs, erythro-myeloid progenitors; dHSCs, definitive hematopoietic stem cells; HSPCs, hematopoietic stem and progenitor cells; P-Sp, paraaortic splanchnopleura.

The blood system is developed at early stages of ontogeny to support the growing embryo at the initiation of heartbeat. Intriguingly, these first primitive blood cells develop independently of HSCs in the yolk sac at murine embryonic day (E)7 and consist of erythrocytes, megakaryocytes, and macrophages, critical for the embryo’s basic needs (Palis, 2016). Prior to the emergence of HSCs, a second wave of erythromyeloid progenitors (EMPs) initiates in the yolk sac around E8, and progenitors with lymphoid potential emerge both in the yolk sac and embryo proper at around E9.5 (Yoshimoto et al., 2011, 2012; Boiers et al., 2013; Palis, 2016; Ghosn et al., 2019). Definitive HSCs (dHSCs) responsible for life-long maintenance of the blood system are first observed at E10.5 in the mouse system, arising in the aorta–gonad–mesonephros (AGM) region (Dzierzak and Bigas, 2018). dHSCs arise alongside HSC-independent progenitors from endothelial cells lining the dorsal aorta in a process known as endothelial to hematopoietic transition (EHT; Ottersbach, 2019; Zhu et al., 2020). EHT is additionally involved in the formation of EMPs and likely also lympho-myeloid progenitors in the yolk sac (Frame et al., 2016; Palis, 2016). Circulating HSCs colonize the fetal liver (FL) approximately at E12 and undergo a massive expansion phase before finally migrating to the bone marrow at E17.5, the main site of hematopoiesis during the life time of an individual (Ema and Nakauchi, 2000; Gao et al., 2018). Of note, the FL niche harbors both HSC-dependent as well as HSC-independent progenitors during development, adding to heterogeneity and complexity (Schematic overview of hematopoietic development is shown in Figure 1).

**FIGURE 2:**
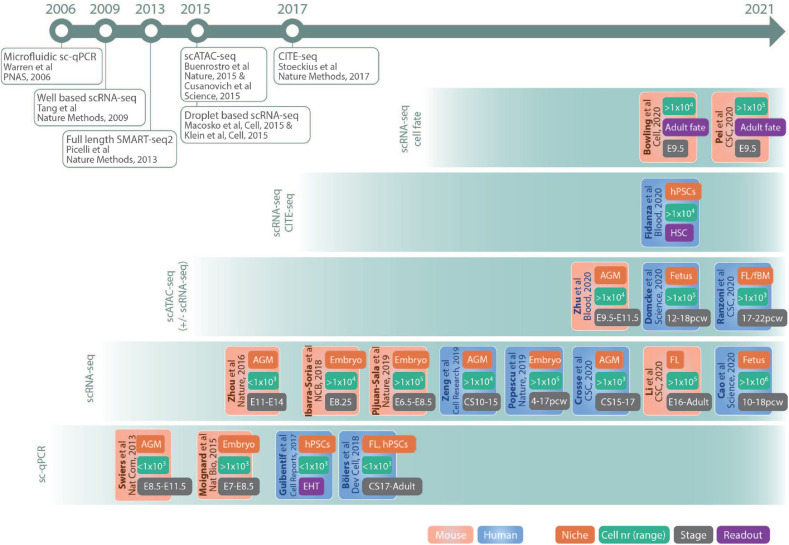
Schematic drawing of key studies highlighted in the text. The references are grouped in panels according to the main method used and listed according to publication date. The color code of the boxes indicates if the study was performed in mouse (*red*) or human (*blue*). The different tags in the boxes indicates main fetal origin of cells investigated (*niche*, *red*), nr of cells sequenced (*cell nr*, *green*), approximate developmental age (*stage*, *gray*), and readout (*purple*). Publications of important single-cell methods are listed in the timeline at the top. AGM, aorta–gonad–mesonephros; fBM, fetal bone marrow; CS, Carnegie stage; E, embryonic day; EHT, endothelial to hematopoietic transition; FL, fetal liver; hPSCs, human pluripotent stem cells; pcw, postconceptual weeks; HSCs, hematopoietic stem cells. Abbreviations Journals: CSC, Cell Stem Cell; Dev Cell, Developmental Cell; NCB, Nature Cell Biology; Nat Bio, Nature Biotechnology; Nat Com, Nature Communications.

This review summarizes the impact of single-cell genomics on hematopoietic ontogeny research, outlining the suitability of different methods for the investigation of various processes. It focuses on representative and key studies (outlined in Figure 2) in mouse and human that illustrate how technological advances offer a possibility to resolve previously elusive conceptual questions regarding embryonic and fetal hematopoiesis, as well as discusses future challenges and possibilities.

## Single-Cell qPCR Analysis Shed Light on Emerging dHSCs

The development of microfluidic chips allowing for simultaneous quantitative PCR (qPCR) reactions of hundreds of single cells at nanoliter volumes paved the way for the single-cell (sc)-genomic era of the last decade (Warren et al., 2006; Spurgeon et al., 2008). Compared to more recent scRNA-sequencing (scRNA-seq) methods, sc-qPCR analysis offers higher sensitivity and specificity but substantially lower throughput both in cell numbers and features. Additionally, the targeted approach of sc-qPCR demands a high level of prior knowledge of the test cells for efficient primer-panel design. Thus, early single-cell experiments in embryonic hematopoiesis focused on EHT, where hematopoietic stem and progenitor cells (HSPCs) emerge from the hemogenic endothelium (HE) of the dorsal aorta. dHSCs are rare in the early embryo (Kumaravelu et al., 2002), and consequently, the target populations are small, while the gene targets are distinct (endothelial/hematopoietic) and well described. Using a green fluorescent protein (GFP)-reporter mouse model for hematopoietic-associated *Runx1* activity, Swiers et al. (2013) isolated and performed sc-qPCR analysis on a total of 803 cells, from E8.5 to E11.5. Despite using a primer panel consisting of only 18 established endothelial or hematopoietic gene markers, it could be shown that single GFP positive cells lost endothelial potential around E9.5 and gradually activated a hematopoietic molecular signature. Thus, single-cell analysis made it possible to reveal that the endothelial wall consists of HE that is molecularly specified toward hematopoietic fate already 2 days before definitive hematopoiesis emerges.

In a similar approach, the formation of primitive blood was investigated at four different timepoints from E7.0 to E8.5 (Moignard et al., 2015). Here, a Runx1-GFP reporter mouse was used in combination with the vascular endothelial growth factor receptor FLK1, to catch early hematopoiesis and mesoderm with hematopoietic potential, respectively. In total, five populations and almost 4,000 single cells were analyzed against primers targeting relevant transcription factors. The high number of cells from a putative hierarchy of cell states allowed for the visualization of trajectories for blood development and the bifurcation of blood and endothelium. The focus on transcription factors, furthermore, made possible the identification of regulatory networks that specify early blood formation, specifically *Sox7*, *Hoxb4*, and *Erg* factors. Thus, the sensitivity of sc-qPCR is powerful for mechanistic insights into developmental processes when combined with high cell numbers and carefully selected primer panels.

Similar to mouse, HSCs in human development also emerge through EHT in the AGM region, and from around Carnegie stage (CS), 13 (day 27) hematopoietic clusters can be seen in the dorsal aorta (Ivanovs et al., 2017). Understanding of gene regulatory networks behind EHT and how dHSCs emerge in human is of clinical relevance to generate functional HSCs from human pluripotent stem cells (hPSCs). However, conventional analysis of dHSCs generation within the human embryo is challenging due to a combination of limited access to material as well as rarity and heterogeneity of relevant cell populations.

Using the power of single-cell technologies, Guibentif et al. (2017) explored the EHT process in hPSCs differentiated toward blood using sc-qPCR analysis. CD34-positive cells were index sorted on day 10 of differentiation, a timepoint when HE cells, HSC-like populations, as well as a mixture of EHT-related cells are present in the culture. A total of 437 cells and a panel of 91 genes, related to the EHT process, were analyzed. Based on molecular signatures, a rare but distinct population of immediate precursors to hematopoietic progenitors, coexpressing endothelial and key hematopoietic genes, was observed. Combining molecular and cell surface (index) data allowed for prospective isolation of these EHT cells and subsequent functional analysis, which revealed that hematopoietic potential preceeds complete downregulation of the endothelial program.

Taken together, although limited in throughput and targeted in nature, the high sensitivity and accuracy of sc-qPCR analysis has been useful for answering questions related to transcription factor activity and gene regulatory networks. As such, sc-qPCR methods could still complement current less-sensitive protocols for global sc-genomics.

## Using Single-Cell RNA-Seq to Study Organogenesis and Emerging dHSCs

The advent of scRNA-seq represents an advancement of single-cell genomics from targeted to global, unsupervised gene expression analysis (Tang et al., 2009; Picelli et al., 2013; Klein et al., 2015; Macosko et al., 2015; See et al., 2018). Similar to sc-qPCR, early scRNA-seq studies focused on preHSCs in the mouse embryo. Using flow cytometry, a total of approximately 100 endothelial cells and different types of preHSCs from the AGM region were purified at E11 as well as HSCs from FL at E12 and E14 (Zhou et al., 2016). Despite the low throughput, this approach, together with the possibility to measure expression of >5,000 genes/cell, allowed for investigation of the dynamic change in gene expression, as cells transform from endothelial cells to functional HSCs. Importantly, the preHSC population was found to be molecularly heterogeneous, highlighting the need for high-throughput single-cell methods. A more comprehensive transcriptional map of EHT in E9-11 AGM mouse embryos as well as gene regulatory networks and trajectories involved in the EHT process have recently been published (Baron et al., 2018; Bergiers et al., 2018; Zhu et al., 2020).

Recently, analysis of HSPCs formation by scRNA-seq methods has been extended to human embryos. As in the mouse system, there are only a few known surface markers that enrich for early preHSCs. However, exploiting the high-throughput capacity of droplet-based sequencing (10xGenomics) together with the low-throughput but more sensitive well-based RNA-seq protocol, the heterogeneity of the dorsal aorta in the human AGM region in early CS10–15 embryos could be dissected, resulting in the visualization of a landscape for HSC generation in the AGM region (Zeng et al., 2019). Importantly, hemogenic endothelial cells was observed and identified as positive for the cell surface marker CD44. These were primed toward the hematopoietic lineage coexpressing endothelial genes (e.g. *CDH5*, *SOX7*, and *ERG*) and hematopoietic transcription factors (e.g. *RUNX1*, *MYB*, and *ANGPT1*).

A marked feature of HSC emergence is that it occurs in discrete anatomical sites, specifically in clusters at the ventral wall of the dorsal aorta (Ivanovs et al., 2017). To characterize the HSC-promoting mechanisms of this niche, spatial transcriptomics was recently performed using laser capture dissection coupled with RNA sequencing (LMO-seq) at CS15–17 (Crosse et al., 2020). Additionally, the transcriptome of 2300 single cells from the dorsal aorta were examined by scRNA-seq. Together, this approach allowed for defining the molecular mechanisms behind EHT within the aortic niche as well as the identification of cell–cell interactions. Within the identified ventrally enriched signaling pathways, a novel factor, endothelin, secreted in the ventral domain of dorsal aorta, was found to promote the development of aortic clusters. This finding was subsequently validated by the authors in both mouse and human models as a potential important factor to promote formation of HSCs *ex vivo* (Crosse et al., 2020).

The increasing throughput capacity of single-cell transcriptomics has enabled studies of whole embryos to address conceptual questions regarding organogenesis, overcoming previous confounding factors such as heterogeneity, lack of cell-type-specific surface markers, and rare populations. Investigating whole mouse E8.25 embryos using droplet-based sequencing, >20,000 single cells could be characterized and 20 major cell types identified, including the brain, gut, and blood precursors. Focusing on the endothelial cell transitioning to blood, *Alox5*, involved in leukotriene production, was found to be expressed in the transition to EMP, and further functional validation found leukotriene to promote development of hematopoietic progenitors (Ibarra-Soria et al., 2018). These data represented a snapshot of the transcriptome during organogenesis but was later followed by other, time-course studies providing a cell atlas of both mouse and human blood development (Pijuan-Sala et al., 2019; Cao et al., 2020). In the study by Pijuan-Sala et al. (2019), early developmental stages from E6.5 to E8.5 were explored. Furthermore, a transcriptional map from embryo chimeras of *Tal^–/–^*, an important transcription factor in hematopoietic development, was created to characterize the defect in early mesoderm specification (Pijuan-Sala et al., 2019). Cao et al. compared hematopoiesis at different sites in human fetal development by investigating different organs. The heterogeneity map generated was similar to what has been observed in scRNA-seq studies using other methods (Popescu et al., 2019; Bian et al., 2020), and interestingly, the human data set could be integrated and compared with a mouse embryonic cell atlas (Cao et al., 2020).

An important goal in regenerative medicine is to generate functional HSCs from hPSCs. EHT includes the formation of both HSC-dependent as well as HSC-independent progenitors. Thus, which progenitors produced by the hPSC culture and the corresponding embryonic wave it recapitulates is difficult to state. It is also unknown if *de novo* HSCs are formed. The holistic, unsupervised, and high-throughput data generated by scRNA-seq allow for defining the PSC-differentiation process, including cell hierarchies, molecular regulation, and genetic networks, as well as for resolving heterogeneity of HSPCs formed in the culture (Angelos et al., 2018; Han et al., 2018). Recently, scRNA-seq followed by trajectory analysis revealed the cellular heterogeneity and differences between hPSCs differentiated toward blood and fetal HSCs (Fidanza et al., 2020). Furthermore, candidate surface markers with the potential to prospectively isolate distinct populations within the differentiation hierarchy were identified. Lately Cellular Indexing of Transcriptomes and Epitopes by Seq (CITE-seq; Stoeckius et al., 2017), a modified version of scRNA-seq, has been introduced where cells are stained with antibodies coupled to unique oligonucleotides that are subsequently included in the sequencing library, thus making possible direct correlation of immunophenotype and transcriptome. CITE-seq was applied to validate the cell surface markers identified by scRNA-seq in Fidanza et al. (2020), comprehensively defining the cellular and immunophenotypic hierarchy of hPSC differentiation *in vitro*. Importantly, by performing machine learning and comparing the data with published single-cell transcriptome data from the human embryo (Popescu et al., 2019), distinct cell types could be identified in the *in vitro* data set. Studies like this will help in identifying factors and differences that can improve culture conditions to generate functional HSCs for future clinical applications.

## Cell Hierarchies and Trajectories Resolved by Single-Cell Genomics

Adult murine hematopoiesis is viewed as a textbook example of a hierarchical structure, with the self-renewing, multipotent HSC on top of a range of increasingly lineage-committed hematopoietic progenitors (Kondo et al., 1997; Akashi et al., 2000). The hematopoietic hierarchy is constantly revised for new progenitors and complexity, while comparable cell states are recognized in human hematopoiesis (Doulatov et al., 2012; Jacobsen and Nerlov, 2019). Even when progenitor populations are carefully isolated based on expression of multiple cell-surface markers, high-throughput scRNA-seq experiments have revealed a heterogeneity resembling a differentiation continuum rather than a stepwise commitment through defined intermediate cell states (Nestorowa et al., 2016; Velten et al., 2017; Buenrostro et al., 2018; Laurenti and Gottgens, 2018).

Lineage commitment and cell fate decisions during ontogeny are less defined. However, clear differences compared to adult have been observed. Distinct fetal progenitors have been identified both in mouse and human, whereas certain immune cells are formed mainly during fetal development (Boiers et al., 2013; Notta et al., 2015; Beaudin et al., 2016; Ghosn et al., 2019). Importantly the different hematopoietic waves overlap in time as well as niche, adding to the heterogeneity. Popescu et al. created a transcriptional map of the human blood system during development, covering a time window from 4 to 17 postconceptual weeks (pcw) and impressively investigated 140,000 FL cells, as well as immune cells from other tissues like skin and kidney (Popescu et al., 2019). In total 27 different cell states were identified, including nonhematopoietic cells like hepatocytes. Hematopoietic lineage trajectory showed an HSC/multipotent progenitor (MPP) cluster differentiating toward lymphoid, myeloid, and megakaryocyte–erythroid–mast cell (MEM) progenitor populations, where HSCs/MPPs were situated at the branching point of lineage commitment, with early transcriptome priming toward all the different lineages (Popescu et al., 2019). This extensive source of data has already been used as a reference material for later studies (Cao et al., 2020; Fidanza et al., 2020).

Understanding lineage potential and commitment during ontogeny is of particular interest, as some mutations that give rise to childhood leukemia occurs *in utero*, resulting in children being more prone than adults to develop acute lymphoblastic leukemia (ALL), particularly B-ALL (Greaves, 2018). In an attempt to identify differences in fetal and adult lymphoid lineage commitment, a fetal progenitor expressing interleukin 7 receptor (IL7R) was characterized in early human development (Boiers et al., 2018). Taking advantage of the sensitivity of sc-qPCR analysis, lineage-affiliated genes were investigated and CD19^–^IL7R^+^ progenitors observed to comprise a unique lineage program, coexpressing myeloid and B-lymphoid-associated genes at the single-cell level. This developmentally restricted progenitor is interesting, as it represents a possible target childhood-leukemia-initiating cell emerging *in utero* (Greaves, 2018).

Transcriptional control of gene expression involves interaction of transcription factors with cis-regulatory elements such as promoters and enhancers. A limitation of scRNA-seq is the high-dropout levels resulting in impaired detection of low-expressed cell-type-specific transcription factors likely restricting identification of key regulators of cell function and priming. Additionally, a recent study utilizing transcribed barcodes together with scRNA-seq revealed that scRNA-seq alone could not fully resolve subtle changes involved in early hematopoietic differentiation (Weinreb et al., 2020), suggesting that other complementary methods could be used to further dissect the intricate dynamics of the process. Lately, assay for transposase-accessible chromatin using sequencing (ATAC-seq) was developed and quickly adapted for single-cell analysis (Buenrostro et al., 2015; Cusanovich et al., 2015). ATAC-seq measures chromatin accessibility, and the resulting data provide epigenetic information including active transcription factor binding sites, promotor as well as distal element usage, and accessible enhancers (Buenrostro et al., 2015; Cusanovich et al., 2015; Corces et al., 2016). ATAC-seq interrogation of distal elements have also been suggested to be superior in cell-type classification compared to RNA-seq (Corces et al., 2016). Thus, scATAC-seq provides a complementary viewpoint of heterogeneity and differentiation to scRNA-seq.

By means of a barcoded indexing approach, Domcke et al. (2020) were able to perform scATAC-seq on ∼8 × 10^5^ single cells generating a cell atlas of embryonic gene regulation covering 15 organs and 59 human fetal samples ranging between 12 and 18 pcw. These data include a comprehensive analysis of blood differentiation over time and also shows that hematopoietic cell types are similar across fetal organs (Domcke et al., 2020).

Ranzoni et al. (2020) explored lineage priming and commitment in human ontogeny at single-cell transcriptome as well as single-cell epigenetic level. Focusing on later stages of FL hematopoiesis (17–22 pcw) and including fetal BM from the same donor, HSCs and progenitors were sorted and RNA-seq performed according to SMART-seq2 (Picelli et al., 2013). The immunophenotype of the sorted populations could thus be linked to the transcriptome data. New surface markers were identified from the most highly expressed genes within the HSC/MPP cluster and used to improve purity of prospectively isolated populations. Moreover, chromatin accessibility was linked with lineage commitment, and integration of scRNA-seq and scATAC-seq data revealed high correlation between cell types. However, within the HSC/MPP population, a change in chromatin accessibility was observed before the onset of lineage commitment, indicative of priming at the chromatin level. The study highlights that lineage commitment and cell fate choice may occur at multiple levels and thus the benefit of combining chromatin accessibility with transcriptome analysis (Buenrostro et al., 2018; Weinreb et al., 2020).

## Describing Fetal to Adult Transition With Single-Cell Methods

Fetal and adult HSCs differ molecularly, in lineage potentials, as well as in cell cycle activity (Ema and Nakauchi, 2000; Bowie et al., 2006; Kim et al., 2007; Yuan et al., 2012; Beaudin et al., 2016; Popescu et al., 2019). Fetal HSCs are more self-renewing and proliferating than the adult counterpart, not adapting an adult-like state until around 3 weeks after birth (Bowie et al., 2006). How this transition from fetal to adult is regulated was recently investigated in mouse development, where scRNA-seq was combined with bulk ATAC-seq and ChIP-seq to investigate the transcriptional and epigenomic landscapes at different timepoints from E16 to adult (Li et al., 2020). The postnatal samples clustered separately from fetal and adult samples, indicating a gradual switch toward adult state. Furthermore, looking at fetal and adult identity scores, the changes in fetal and adult gene expressions were found to be uncoordinated causing heterogeneity. Of note, an increase in interferon just before birth proceeded the transition and was shown to induce adult transcriptional programs and made the cells vulnerable to transformation. This might have importance in our understanding of childhood leukemia, where some mutations have been shown to occur *in utero* (Greaves, 2018).

A conceptual question in fetal hematopoiesis is whether HSCs acquire a lineage preference during fetal life that is retained in adulthood. Typically, lineage trajectories are computationally inferred by the similarity of cells and their relative distance to each other and thus does not necessarily reflect the clonal relationship between cells (Weinreb et al., 2020). To link transcriptome with lineage fate, transcribable inherited marks can be introduced in the genome of the target cell allowing for the progeny of that cell to be traced. This allows for investigation of the clonal relationships of cells in conjuncture with their transcriptomic identity. Such approaches could answer lineage relationship during development and link it to gene expression signature. New techniques are rapidly evolving, where transcriptome and lineage tracing can be combined, using for instance transcribed barcodes introduced into the genome by CRISPR-Cas9. Using these kinds of methods, developmental lineage relationships during gestation in zebrafish, and later also mouse, have been investigated (Alemany et al., 2018; Kester and van Oudenaarden, 2018; Chan et al., 2019). Further development of the methods by introducing an inducible Cas9 enabled labeling at different developmental time points (Bowling et al., 2020). The study is a clear proof of concept, and lineage bias of fetal HSCs was also investigated. By labeling embryos at E9.5, when dHSCs are formed, labeled clones and their progeny could be traced in adult mice together with the transcriptome of HSCs and progenitors. Investigated clones did not show any significant lineage preference; however, they were unevenly distributed across bones, showing a bias in localization within the niche. A similar strategy, utilizing a *PolyloxExpress* barcoding system, has also been used to study cell fate of fetal HSCs in adult mice (Pei et al., 2020). Although only in their infancy, these state-of-the-art methods have already shown great promise of deciphering the earliest fate decisions. Additionally, with versatile induction of tracing, they could be applied to different developmental timepoints and genetic backgrounds to study numerus biological questions. However, these models are depending on barcode diversity as well as high labeling efficiency. Furthermore, detection levels of the unique barcodes need to be high (Wagner and Klein, 2020). By using a DNA barcode library, cells from diverse models, including human, can be labeled; however, the cells cannot be investigated *in situ* (Weinreb et al., 2020). Other methods evolving for human settings are to use somatic mutations, which can be detected with whole genome sequencing, and used to trace history of cells. By amplifying single cells in culture, enough material can be obtained for sequencing and subsequent tracing of different mutations to draw pyogenic trees. It has been shown that such approaches can trace cells back as early as to gastrulation (Lee-Six et al., 2018), and indeed, a recent study is using somatic mutation tracing to understand lineage relationships during ontogeny (Chapman et al., 2020).

## Concluding Remarks

The evolution of sc-genomic techniques has opened new possibilities for understanding developmental hierarchies and differentiation landscapes. Future studies are likely to make further use of the current efforts to increase cell throughput, such as Scifi-seq (Datlinger et al., 2019). Together with multiomics, such as ASAP-seq (Mimitou et al., 2020), ECCITE-seq (Mimitou et al., 2019), and combined scATAC/RNA-seq (10xGenomics), these protocols are important for developmental hematopoiesis, as they will improve detection of rare and embryo-specific cell types as well as make it possible to integrate cell fate, transcriptomics, proteomics, and epigenetic changes at single-cell resolution also when tissue is sparsely available, such as during embryonic development. These techniques will also be important from a disease perspective, where rare mutations in congenital immune and blood disorders can be investigated and compared to existing human developmental maps. In addition, many mutations that lead to childhood ALL occurs *in utero*, creating a preleukemic state. Integrating mutational status of a cell with transcriptional status may allow for identification of leukemic stem cells in a heterogeneous leukemic bulk sample and thus makes it possible to identify and characterize these cells and may allow for the development of new treatments. Undeniably, sc-genomics will continue to have critical impact on developmental hematopoiesis research and subsequently for our understanding of congenital immune and blood disorders, childhood leukemia, cell-replacement therapy, and for regenerative medicine as a whole.

## Author Contributions

GK and CB: writing (original draft). All author: writing (review and editing), visualization, and final approval of the submitted manuscript.

## Conflict of Interest

The authors declare that the research was conducted in the absence of any commercial or financial relationships that could be construed as a potential conflict of interest.
